# Clinical application, potential pharmacological targets and quality marker prediction of a TCM formulation used (Shenling Baizhu San) in the treatment of respiratory diseases

**DOI:** 10.3389/fphar.2025.1575903

**Published:** 2025-05-29

**Authors:** Zu Gao, Tong Wang, Liwen Fu, Qiaolan Wu, Yuan Wang, Zhichun Wu, Guangying Lu, Chunxue Ou, Haijun Zhao, Huayun Yu

**Affiliations:** ^1^ College of Traditional Chinese Medicine, Shandong University of Traditional Chinese Medicine, Jinan, China; ^2^ College of Nursing, Shandong University of Traditional Chinese Medicine, Jinan, China; ^3^ Innovative institute of Chinese Medicine and Pharmacy, Shandong University of Traditional Chinese Medicine, Jinan, China; ^4^ College of Medicine, Shandong University of Traditional Chinese Medicine, Jinan, China; ^5^ Shandong Co-Innovation Center of Classic TCM Formula, Shandong University of Traditional Chinese Medicine, Jinan, China

**Keywords:** Shenling Baizhu San, respiratory diseases, clinical application, pharmacological mechanisms, quality markers

## Abstract

Shenling Baizhu San (SLBZS) is a formulation of traditional Chinese medicine (TCM) recorded in the Song Dynasty medical book *Taiping Huimin Heji Jufang* (AD 1078–1085). It comprises eleven herbs: Ginseng Radix Et Rhizoma, Atractylodis Macrocephalae Rhizoma, Poria, Dioscoreae Rhizoma, Nelumbinis Semen, Coicis Semen, Lablab Semen Album, Amomi Fructus, Platycodonis Radix, Glycyrrhizae Radix Et Rhizoma, and Jujubae Fructus. SLBZS has been employed for over 900 years in the treatment of pulmonary and gastrointestinal disorders because of its qualities that enhance spleen function, tonify the lungs, supplement qi, and mitigate diarrhoea. This study meticulously examined and synthesised the clinical relevance and pharmacological mechanisms of SLBZS, concentrating on respiratory diseases, in response to the increasing volume of clinical data about SLBZS. Meanwhile, according to the five principles of Q-marker determination, including quality transmission and traceability, metabolites specificity, formula compatibility environment, association between metabolites and effectiveness, metabolites measurability, the potential quality markers (Q-markers) that SLBZS in the treatment of respiratory diseases were predicted. This study will provide additional clinical research, clarify pharmacological mechanisms, and set quality control criteria for SLBZS in the treatment of respiratory diseases.

## 1 Introduction

Shenling Baizhu San (SLBZS) originated from the medicinal book *Taiping Huimin Heji Jufang* in the Northern Song Dynasty. As a formula of traditional Chinese medicine (TCM), it is composed of Renshen (Ginseng Radix Et Rhizoma), Baizhu (Atractylodis Macrocephalae Rhizoma), Fuling (Poria), Shanyao (Dioscoreae Rhizoma), Lianzi (Nelumbinis Semen), Yiyiren (Coicis Semen), Baibiandou (Lablab Semen Album), Sharen (Amomi Fructus), Jiegeng (Platycodonis Radix), Gancao (Glycyrrhizae Radix Et Rhizoma) and Dazao (Jujubae Fructus) (The Pharmacopoeia of the People’s Republic of China, 2020) (Table 1). SLBZS promotes useful qi and supports the spleen, alleviates dampness, and possesses anti-diarrhoeal properties. It is typically employed for diarrhoea resulting from spleen deficiency and dampness, as well as for lung qi deficit, phlegm dampness, cough, and asthma. The recipe incorporates Renshen, Baizhu, and Fuling to enhance qi and spleen function, eliminate moisture, and facilitate transportation. Shanyao and Lianzi enhance spleen and qi, possess astringent properties for the intestines, and serve as antidiarrheal agents; Baibiandou and Yiyiren effectively eliminate dampness and mitigate diarrhoea; Sharen works to eliminate dampness, promote qi circulation, and harmonise the stomach. SLBZS may serve as adjunctive medications for addressing gastrointestinal disorders, including ulcerative enteritis and persistent diarrhoea in clinical settings (Chen et al., 2022; Wang et al., 2022). Jiegeng facilitates lung qi circulation, alleviates obstructions, and regulates qi flow, rendering SLBZS effective in treating respiratory conditions such as chronic obstructive pulmonary disease (COPD) and bronchial asthma (Mao et al., 2021; Huang et al., 2023). The metabolites of SLBZS are intricate, particularly regarding the pharmacological mechanisms and quality marker metabolites in the treatment of respiratory disorders, which remain ambiguous and require more clarification.

**TABLE 1:** Botanical drugs of SLBZS.

Botanical name	English name	Local name	Part used	Fixed ratio
*Panax ginseng* C.A.Mey	Ginseng Radix Et Rhizoma	Renshen	Rhizome and Root	15
*Atractylodes macrocephala* Koidz	Atractylodis Macrocephalae Rhizoma	Baizhu	Rhizome	15
*Poria cocos* (Schw.) Wolf	Poria	Fuling	Sclerotia	15
*Dioscorea opposita* Thunb	Dioscoreae Rhizoma	Shanyao	Rhizome	15
*Nelumbo nucifera* Gaertn	Nelumbinis Semen	Lianzi	Seed	9
*Coix lacryma-jobi* L. var. *Mayuen* (Roman.) Stapf	Coicis Semen	Yiyiren	Seed	9
*Dolichos lablab* L	Lablab Semen Album	Baibiandou	Seed	12
*Amomum villosum* Lour	Amomi Fructus	Sharen	Fruit	6
*Platycodon grandiflorum* (Jacq.) ADC.	Platycodonis Radix	Jiegeng	Root	6
*Glycyrrhiza uralensis* Fisch	Glycyrrhizae Radix Et Rhizoma	Gancao	Rhizome and Root	10
*Ziziphus jujuba* Mall	Jujubae Fructus	Dazao	Fruit	7.5

The metabolites of TCM formulae are essential for clarifying their pharmacological effects. Previous research has comprehensively documented the aqueous extract of a specific TCM botanical medication in SLBZS, along with the metabolites found in the serum (Wang et al., 2025; Wu et al., 2024; Han et al., 2025). Specific metabolites have exhibited beneficial effects on respiratory diseases (Li B. S. et al., 2021; Zhongyin et al., 2022; Xu et al., 2025). Nonetheless, there is inadequate evidence regarding the metabolites of SLBZS and the specific active metabolites that facilitate its therapeutic effects on respiratory illnesses. Quality markers (Q-markers) are active metabolites that signify the pharmacological properties, effectiveness, and quantifiability of TCM botanical drugs. This will act as a reference for the quality control of TCM botanical drugs and formulae, specifically focusing on metabolites with potential pharmacological effects, thereby becoming crucial for the development and application of TCM botanical drugs and formulae (Wu et al., 2024).

Recent years have seen the therapeutic efficacy of SLBZS validated by substantial foundational research and clinical trials, with the development of a broad array of qualitative and quantitative procedures for its chemical analysis and quality control. Nevertheless, these research findings have not been thoroughly synthesized. This research analyses the clinical application and pharmacological effects of SLBZS, utilizing the five principles of Q-marker to predict potential Q-markers of SLBZS in the treatment of respiratory illnesses. This study aims to improve the quality control standards of TCM botanical drugs and formulae, while providing insights and a basis for the ongoing development and implementation of SLBZS.

## 2 Methods of data acquisition

This study utilises primary literature obtained from the PubMed, Web of Science, and China National Knowledge Infrastructure (CNKI) databases. The search keywords are “Shenling Baizhu San”, “Respiratory System”, “Clinical Application”, “Pharmacological mechanism”, “metabolites”, “Ginseng Radix Et Rhizoma”, “Atractylodis Macrocephalae Rhizoma”, “Poria”, “Dioscoreae Rhizoma”, “Nelumbinis Semen”, “Coicis Semen”, “Lablab Semen Album”, “Amomi Fructus”, “Platycodonis Radix”, “Glycyrrhizae Radix Et Rhizoma”, “Jujubae Fructus” and their combinations. The search ended on 31 January 2025, without any preceding time limitations established. The criteria for admission and disqualification were delineated as follows: 1) Clinical application of SLBZS or its modified variants in the treatment of respiratory disorders; 2) Pharmacological mechanisms associated with SLBZS or its modified variants in managing respiratory disorders; 3) Pharmacological mechanisms of the herbal metabolites found in SLBZS for respiratory disorder treatment; 4) Identification and quantitative assessment of metabolites in SLBZS. Reviews, meta-analyses, and case reports were excluded from the research. In the screening process, we initially reviewed titles and abstracts to find relevant studies that fit the inclusion criteria, followed by a thorough analysis of full-text publications, resulting in the inclusion of 64 papers.

## 3 Clinical application of SLBZS in respiratory diseases

### 3.1 Clinical application of SLBZS in COPD

COPD is a common respiratory disorder caused by alveolar or airway irregularities arising from continuous exposure to significant levels of toxic gases or particles. It is chiefly characterised by persistent airway obstruction, associated with elevated rates of disability and mortality (Wang et al., 2024a). A randomised controlled trial (Shi, 2024) assigned 104 patients with stable COPD to a control group and an observation group in a 1:1 ratio. The control group was administered tiotropium bromide powder inhalation, while the observation group received modified SLBZS alongside the control group’s treatment. The findings indicated that modified SLBZS might markedly decrease the COPD assessment test (CAT) score and enhance pulmonary function metrics, including forced expiratory volume in one second (FEV1), forced vital capacity (FVC), and the FEV1/FVC ratio. A randomised controlled trial involving 78 COPD patients with lung-qi deficit (He, 2024) revealed that the combination of modified SLBZS significantly improved the overall effective treatment rate, FVC, FEV1, and FEV1/FVC in comparison to the administration of budesonide and formoterol inhalation powder alone. The effect was significantly superior to that of inhaling powder aerosol alone. A study by Zhang et al. (2023) demonstrated that the combination of SLBZS and salmeterol improved the overall efficacy of COPD treatment and improves FVC, FEV1, and FEV1/FVC ratios compared to salmeterol alone. Concurrently, it can improve arterial oxygen saturation (SaO2), arterial partial pressure of oxygen (PaO2), arterial partial pressure of carbon dioxide (PaCO2), mucin 5AC (MUC5AC) in induced sputum, matrix metalloproteinase-9 (MMP-9), neutrophil elastase (NE), serum hypersensitive C-reactive protein (hs-CRP), interleukin-6 (IL-6), and plasma Brain Natriuretic Peptide (BNP) levels, exhibiting superiority compared to salmeterol alone.

### 3.2 Clinical application of SLBZS in asthma

Asthma is a common chronic respiratory disorder defined by dyspnoea, chest tightness, wheezing, and other symptoms. There are approximately 300 million sufferers worldwide, with the incidence increasing each year (Li et al., 2020). Huang et al. (2023) found that SLBZS, when combined with salmeterol fluticasone aerosol, substantially mitigated clinical symptoms such as cough and expectoration, while also reducing diurnal peak expiratory flow (PEF), FEV1, FEV1/FVC, and other pulmonary function parameters in patients with chronic persistent bronchial asthma. It also elevated fractional exhaled nitric oxide (FeNO) levels, Asthma Control Test (ACT) scores, and other metrics, while significantly reducing the recurrence rate of bronchial asthma. A distinct randomised controlled trial (Gao, 2022) demonstrated that the combination of SLBZS and fluticasone propionate aerosol markedly reduces the duration and frequency of asthma attacks in children, while also improving clinical symptoms such as wheezing and cough, in comparison to fluticasone propionate aerosol alone. A separate study (Cui and Shang, 2019) demonstrated that modified SLBZS in conjunction with flixxdone might diminish the asthma Control Test (C-ACT) score and enhance FVC, FEV1, peak expiratory flow (PEF), FEV1/FVC, and other pulmonary ventilation function metrics in paediatric asthma patients.

### 3.3 Clinical application of SLBZS in allergic rhinitis

Allergic rhinitis is an allergic disorder of the nasal mucosa, predominantly induced by exposure to allergens in susceptible individuals. It manifests as nasal itching, sneezing, excessive nasal discharge, and nasal congestion, which can easily trigger asthma and sinusitis (Schaefer et al., 2023). Zhao et al. (2024) discovered that the combination of SLBZS and acupuncture alleviates clinical symptoms such as nasal itching, nasal congestion, dizziness, shortness of breath, rhinorrhea, and sneezing in patients with allergic rhinitis. Additionally, it enhances serum levels of interferon-γ (IFN-γ), Cluster of Differentiation 3^+^ (CD3^+^), Cluster of Differentiation 4^+^ (CD4^+^), and the CD4^+^/Cluster of Differentiation 8^+^ (CD8^+^) ratio, while diminishing interleukin-17 (IL-17) and CD8^+^ levels. A distinct randomised controlled trial (Ge and Jiang, 2022) demonstrated that the SLBZS combination, in contrast to standard treatment (loratadine tablets, cetirizine hydrochloride tablets and fluticasone nasal spray), significantly reduced serum concentrations of interleukin-4 (IL-4), interleukin-6 (IL-6), and immunoglobulin E (EIgE), while augmenting levels of INF-γ and Soluble Programmed Death Ligand-1 (sPD-L1) in patients with rhinitis. Clinical symptoms that require alleviation encompass nasal congestion, rhinorrhea, nasal pruritus, sneezing, fatigue, dyspnoea, lethargy, and reduced vocal quality.

### 3.4 Others

SLBZS exhibits a favourable therapeutic impact on pneumonia and recurrent respiratory tract infections. A randomised controlled trial (Li, 2021) indicated that modified SLBZS combined with azithromycin significantly reduced serum concentrations of tumour necrosis factor-α (TNF-α), interleukin-8 (IL-8), IL-6, and CD8^+^, while enhancing levels of CD3^+^ and CD4^+^, thereby promoting lesion absorption and the alleviation of lung rales in paediatric patients with *Mycoplasma* pneumonia. Furthermore, SLBZS combined with massage (Wang, 2024) can significantly improve expectoration, relieve cough, and decrease sputum volume in paediatric patients, while simultaneously lowering inflammatory markers such as IL-4 and TNF-α in sputum. A distinct study (Chen, 2018) revealed that SLBZS combined with pidotimod can reduce serum concentrations of IL-6, TNF-α, procalcitonin (PCT), and cysteinyl leukotriene (Cysl-Ts) in patients with recurrent respiratory tract infections, while also mitigating clinical symptoms such as cough, tonsil hypertrophy, and lung rales.

In summary, SLBZS or modified SLBZS are commonly employed alongside drugs such as tiotropium bromide powder and salmeterol for the treatment of respiratory disorders, exhibiting considerable efficacy (Table 2). Nevertheless, the clinical examination of SLBZS has certain constraints. 1) Methodological constraints are present. Incorporating studies with limited sample sizes (e.g., 78 or 104 cases) may compromise the statistical validity of the findings and elevate the likelihood of a Type II error. 2) The description of the randomisation mechanism is absent. The randomisation grouping lacks a detailed description of the precise mechanism for random sequence generation and allocation concealment, potentially leading to selection bias. 3) Blinding was either defective or omitted. The study did not specify if a double-blind design was implemented, and both patients and assessors were cognisant that subgroups could introduce measurement bias. 4) The dosing specification lacks precision. This study indicates that several physicians may recommend differing dosages for the identical disease. Adequate scientific research on the correlation between dosage and effect are lacking, thereby compromising the efficacy and safety of treatment. 5) Functional testing were conducted individually. Lung function metrics, including FEV1 and FVC, were largely singular and, albeit being statistically significant, did not demonstrate a minimal clinically relevant difference (MCID). 6) Insufficient follow-up. All trials had a follow-up duration of 6 months or less and did not provide evidence on the long-term efficacy and adverse effects, such as herbal liver and kidney damage, of SLBZS. Future investigations in clinical research on TCM will focus on enhancing experimental design, expanding sample inclusion, investigating dose-effect relationships, augmenting the assessment of functional indicators, and prolonging the follow-up period.

**TABLE 2:** Clinical application of SLBZS in respiratory diseases.

No.	Group	Medication (dosage of drug)	Number of people	Treatment effect	The medication time	Reference
1	Observation group	Modified SLBZS was applied on the basis of the control group	52	Total effective rate: 96.15% Prior treatment and after treatment: CAT (17.84 ± 3.28 and 13.32 ± 2.17) TCM syndrome score (15.15 ± 2.68 and 6.76 ± 1.31), FEV1/L (1.44 ± 0.28 and 1.92 ± 0.36), FVC/L (2.18 ± 0.35 and 2.65 ± 0.32), (FEV1/FVC)/% (66.06 ± 7.68 and 77.45 ± 6.31)	One dose per day, divided into two doses, for 3 months	Shi, (2024)
	Control group	Inhaled tiotropium bromide	52	Total effective rate: 88.46% Prior treatment and after treatment: CAT (18.97 ± 3.17 and 15.94 ± 2.48) TCM syndrome score (15.59 ± 2.15 and 8.07 ± 1.48), FEV1/L (1.47 ± 0.17 and 1.70 ± 0.31), FVC/L (2.21 ± 0.34 and 2.44 ± 0.36), (FEV1/FVC)/% (66.52 ± 7.15 and 69.67 ± 5.48)	One inhalation per time (18μg/inhalation), for 3 months	Shi, (2024)
2	Observation group	Modified SLBZS was applied on the basis of the control group	39	Total effective rate: 97.44% Prior treatment and after treatment: FEV1/L (1.19 ± 0.34 and 1.85 ± 0.67), FVC/L (2.13 ± 0.36 and 2.69 ± 0.53), (FEV1/FVC)/% (55.87 ± 5.06 and 68.77 ± 6.33)	One dose per day, divided into two doses, for 2 months	He, (2024)
	Control group	Budesonide formoterol aerosol for inhalation	39	Total effective rate: 79.49% Prior treatment and after treatment: FEV1/L (1.17 ± 0.32 and 1.42 ± 0.51), FVC/L (2.11 ± 0.39 and 2.32 ± 0.47), (FEV1/FVC)/% (55.45 ± 5.09 and 66.21 ± 5.83)	4.5 μg twice a day, for 2 months	He, (2024)
3	Observation group	Modified SLBZS was applied on the basis of the control group	105	Total effective rate: 94.29% Prior treatment and after treatment: FEV1/L (1.32 ± 0.29 and 1.97 ± 0.36), FEV1/% (55.94 ± 12.70 and 69.46 ± 13.80), SaO2/% (81.60 ± 3.52 and 95.37 ± 4.04), PaO2/mmHg (55.96 ± 4.91 and 86.97 ± 5.88), PaCO2/mmHg (54.11 ± 3.61 and 41.82 ± 2.94), hs-CRP/(μg/L) (13.44 ± 1.89 and 6.90 ± 1.31), IL-6/(ng/L) (4.29 ± 1.06 and 2.08 ± 0.61), BNP/(ng/L) (564.15 ± 39.22 and 354.75 ± 22.58), MUC5AC (195.19 ± 22.67 and 87.86 ± 8.24), MMP-9 (172.45 ± 19.22 and 74.44 ± 9.27), NE (1.91 ± 0.47 and 1.33 ± 0.28)	20g twice a day, for 3 months	Zhang et al. (2023)
	Control group	Salmeterol fluticasone inhalation aerosol	105	Total effective rate: 84.76% Prior treatment and after treatment: FEV1/L (1.36 ± 0.19 and 1.58 ± 0.29), FEV1/% (55.75 ± 12.42 and 63.74 ± 13.52), SaO2/% (81.74 ± 3.39 and 91.53 ± 3.75), PaO2/mmHg (55.74 ± 4.87 and 69.58 ± 5.36), PaCO2/mmHg (54.09 ± 3.48 and 46.25 ± 3.26), hs-CRP/(μg/L) (13.27 ± 1.92 and 9.75 ± 1.62), IL-6/(ng/L) (4.31 ± 1.11 and 3.26 ± 0.84), BNP/(ng/L) (567.48 ± 38.46 and 389.42 ± 29.75), MUC5AC (198.16 ± 21.61 and 113.54 ± 12.16), MMP-9 (175.58 ± 19.65 and 93.02 ± 8.23), NE (1.93 ± 0.42 and 1.12 ± 0.22)	50 μg twice a day, for 3 months	Zhang et al. (2023)
4	Observation group	Modified SLBZS was applied on the basis of the control group	60	Prior treatment and after treatment: TCM syndrome score (13.67 ± 1.54 and 2.60 ± 0.52), FEV1/L (1.67 ± 0.38 and 2.87 ± 0.30), (FEV1/FVC)/% (53.15 ± 5.36 and 73.11 ± 5.19), PEF/L/s (2.25 ± 0.42 and 3.64 ± 0.44), FeNO (ppb) (44.01 ± 0.60 and 22.87 ± 0.59), ACT (12.98 ± 0.70 and 22.88 ± 0.57), MDA (μmol/L) (9.28 ± 1.16 and 4.35 ± 0.92), SOD (U/mL) (65.63 ± 10.77 and 89.53 ± 15.86), 8-iso-PG (pg/mL) (151.63 ± 24.37 and 62.86 ± 11.51), GSH-Px (U/mL) (144.55 ± 14.39 and 220.41 ± 18.19), VEGF (266.73 ± 23.38 and 164.91 ± 21.05), TGF-β1 (503.03 ± 31.98 and 415.48 ± 36.79)	One dose per day, divided into two doses, for 12 weeks	Huang et al. (2023)
	Control group	Salmeterol and fluticasone aerosol	60	Prior treatment and after treatment: TCM syndrome score (13.72 ± 1.59 and 6.14 ± 0.71), FEV1/L (1.69 ± 0.35 and 2.31 ± 0.26), (FEV1/FVC)/% (52.87 ± 5.43 and 67.29 ± 5.72), PEF/L/s (2.27 ± 0.38 and 3.09 ± 0.56), FeNO (ppb) (43.95 ± 0.56 and 30.23 ± 0.87), ACT (12.88 ± 0.68 and 17.02 ± 0.66), MDA (μmol/L) (9.22 ± 1.21 and 6.21 ± 0.93), SOD (U/mL) (64.73 ± 11.02 and 82.22 ± 13.61), 8-iso-PG (pg/mL) (153.12 ± 23.79 and 84.32 ± 14.37), GSH-Px (U/mL) (145.20 ± 13.86 and 187.17 ± 17.39), VEGF (268.87 ± 23.92 and 210.69 ± 15.44), TGF-β1 (498.73 ± 32.13 and 415.48 ± 36.79)	Twice a day, 1 inhalation each time, for 12 weeks	Huang et al. (2023)
5	Observation group	Modified SLBZS was applied on the basis of the control group	40	Total effective rate: 97.50% Prior treatment and after treatment: TCM syndrome score (14.11 ± 1.86 and 3.00 ± 1.03), duration of seizures (7.02 ± 1.78 and 4.20 ± 0.98), number of asthma exacerbations (4.78 ± 1.83 and 1.55 ± 0.31)	One dose per day, divided into two doses, for 30 days	Gao, (2022)
	Control group	Fluticasone propionate	40	Total effective rate: 75.00% Prior treatment and after treatment: TCM syndrome score (13.73 ± 2.02 and 5.79 ± 1.66), duration of seizures (7.46 ± 1.64 and 5.90 ± 0.86), number of asthma exacerbations (4.91 ± 1.91 and 2.72 ± 1.22)	50 μg once a day, for 30 days	Gao, (2022)
6	Observation group	Modified SLBZS was applied on the basis of the control group	51	Total effective rate: 84.3% Prior treatment and after treatment: TCM syndrome score (22.84 ± 7.64 and 8.67 ± 6.41), C-ACT (15.35 ± 3.74 and 21.86 ± 4.42), PEF (3.22 ± 1.08 and 4.13 ± 1.22), FEV1/L (1.51 ± 0.50 and 1.92 ± 0.61), FVC/L (1.79 ± 0.60 and 2.59 ± 0.59), (FEV1/FVC)/% (75.71 ± 9.17 and 86.67 ± 6.17)	One dose per day, divided into two doses, for 1 month	Cui and Shang (2019)
	Control group	Fluticasone propionate was inhaled as an aerosol	51	Total effective rate: 72.5% Prior treatment and after treatment: TCM syndrome score (21.86 ± 8.62 and 11.70 ± 7.52), C-ACT (16.17 ± 3.30 and 21.65 ± 3.50), PEF (3.06 ± 1.21 and 3.67 ± 1.20), FEV1/L (1.53 ± 0.56 and 1.71 ± 0.47), FVC/L (1.72 ± 0.67 and 2.13 ± 0.44), (FEV1/FVC)/% (75.77 ± 9.79 and 81.13 ± 8.83)	125 μg once a day, for 1 month	Cui and Shang (2019)
7	Observation group	Acupuncture (Yintang, Yingxiang, Bitong, Fengchi, Shangxing, Hegu) was used on the basis of the control group	50	Total effective rate: 96% Prior treatment and after treatment: IFN-γ (10.12 ± 2.25 and 15.36 ± 4.32), IL-17 (465.32 ± 52.32 and 235.14 ± 35.52), CD3^+^(%) (58.37 ± 3.29 and 69.89 ± 4.59), CD4^+^(%) (33.16 ± 2.13 and 44.04 ± 3.47), CD8^+^(%) (34.26 ± 3.86 and 26.21 ± 2.67), CD4^+^/CD8^+^ (0.97 ± 0.32 and 1.68 ± 0.47)	Once a day for 30 days	Zhao et al. (2024)
	Control group	SLBZS	50	Total effective rate: 74% Prior treatment and after treatment: IFN-γ (10.21 ± 2.26 and 12.42 ± 4.11), IL-17 (465.62 ± 52.49 and 315.36 ± 38.65), CD3^+^(%) (59.54 ± 3.86 and 65.36 ± 4.11), CD4^+^(%) (34.14 ± 2.89 and 39.65 ± 3.21), CD8^+^(%) (33.14 ± 3.54 and 29.23 ± 3.05), CD4^+^/CD8^+^ (1.03 ± 0.35 and 1.36 ± 0.25)	One dose per day, divided into two doses, for 30 days	Zhao et al. (2024)
8	Observation group	Modified SLBZS was applied on the basis of the control group	50	Total effective rate: 68% Prior treatment and after treatment: IFN-γ (28.54 ± 3.57 and 58.76 ± 4.29), IL-4 (59.15 ± 3.26 and 42.76 ± 3.28), IL-6 (12.36 ± 1.28 and 5.93 ± 0.89), IgE(kU/L) (126.53 ± 8.27 and 47.92 ± 4.23), sPD-L1 (ng/mL) (1.72 ± 0.29 and 2.89 ± 0.11)	One dose per day, divided into two doses, for 1 month	Ge and Jiang (2022)
	Control group	Loratadine tablets, Cetirizine hydrochloride tablets, Fluticasone nasal spray	50	Total effective rate:42% Prior treatment and after treatment: IFN-γ (29.76 ± 3.28 and 43.47 ± 4.33), IL-4 (59.83 ± 3.19 and 48.41 ± 3.27), IL-6 (12.45 ± 1.19 and 8.16 ± 0.82), IgE(kU/L) (124.82 ± 8.11 and 73.85 ± 4.51), sPD-L1 (ng/mL) (1.81 ± 0.33 and 2.28 ± 0.12)	0.5 mg (weight ≤30 kg)/time 10 mg (Weight >30 kg)/time, once a day; 2.5 mg (≥3 years old and ≤6 years old)/time, 5–10 mg (>6 years old and ≤12 years old)/time, once a day; 100 μg/time, once a day, for 1 month	Ge and Jiang (2022)
9	Observation group	Modified SLBZS was applied on the basis of the control group	48	Total effective rate: 95.83% Prior treatment and after treatment: TCM syndrome score (25.42 ± 3.35 and 4.19 ± 1.53), CD4^+^ (29.50 ± 3.68 and 38.22 ± 4.89), CD8^+^ (30.15 ± 4.25 and 24.11 ± 3.36), CD3^+^ (51.23 ± 5.13 and 58.12 ± 4.56), TNF-α (ng/L) (29.15 ± 3.76 and 11.43 ± 3.22), IL-8 (ng/L) (27.01 ± 4.01 and 10.03 ± 2.64), IL-6 (ng/L) (21.26 ± 3.02 and 8.79 ± 2.59)	One dose per day, divided into two doses, for 4 weeks	Li, (2021)
	Control group	Azithromycin	45	Total effective rate: 80.00% Prior treatment and after treatment: TCM syndrome score (24.86 ± 4.01 and 8.63 ± 2.57), CD4^+^ (30.04 ± 4.03 and 34.13 ± 5.02), CD8^+^ (29.47 ± 3.97 and 26.09 ± 4.01), CD3^+^ (50.36 ± 4.75 and 55.01 ± 5.12), TNF-α (ng/L) (28.77 ± 3.45 and 15.10 ± 4.09), IL-8 (ng/L) (26.65 ± 3.82 and 13.62 ± 3.25), IL-6 (ng/L) (20.74 ± 2.89 and 11.45 ± 3.13)	Azithromycin 10 mg/kg was given intravenously once a day for 5 days 10 mg/kg (azithromycin dry suspension), once a day, after 3 days of treatment, the drug was stopped for 4 days, and then the treatment was given for 3 cycles	Li, (2021)

## 4 Pharmacological mechanisms of SLBZS in respiratory diseases

**FIGURE 1:**
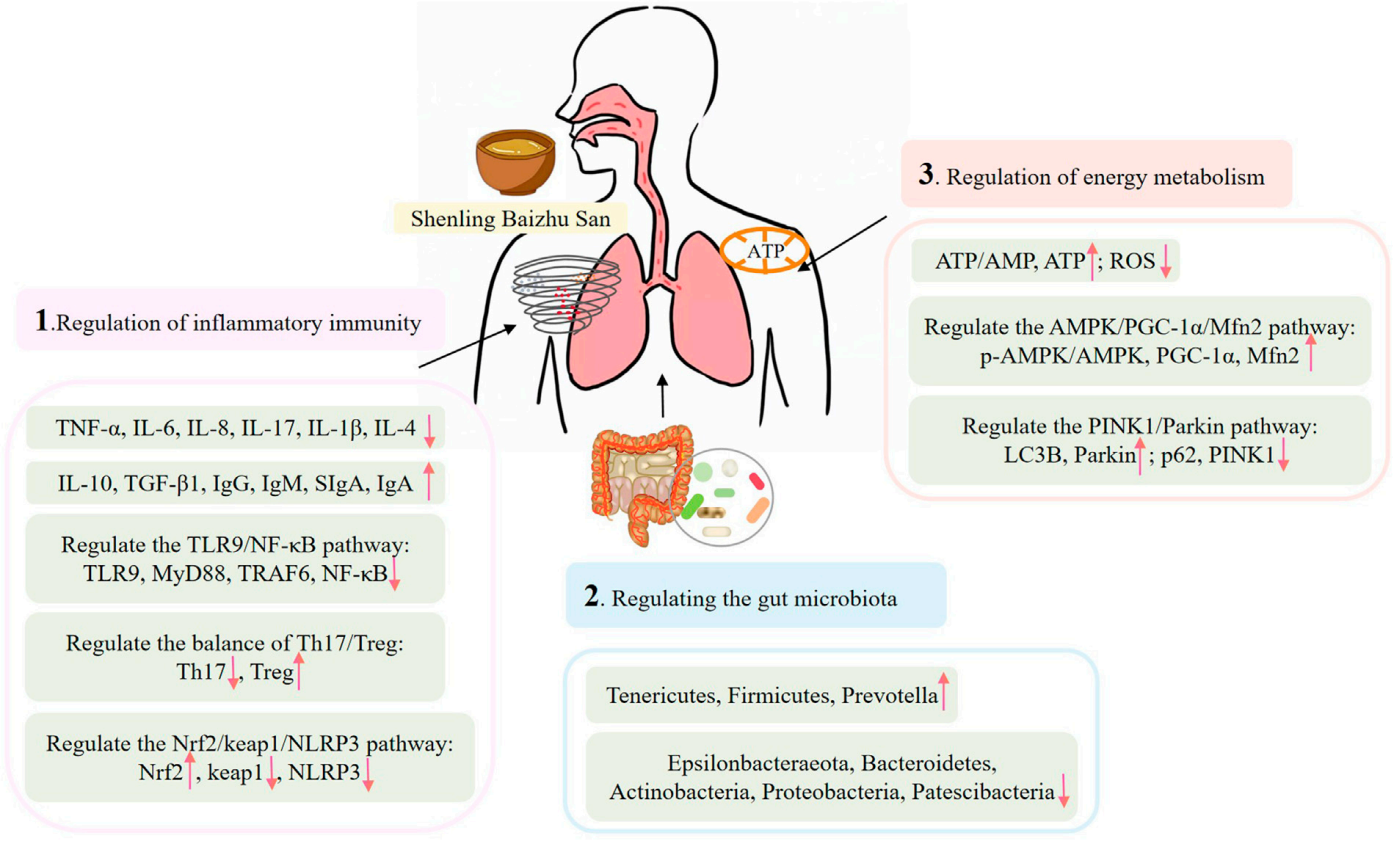
Pharmacological activities of SLBZS.

Pharmacological mechanisms suggest that SLBZS may therapeutically influence COPD, asthma, recurrent respiratory tract infections, pulmonary fibrosis, pneumonia, and other respiratory ailments by inhibiting inflammatory responses in lung tissue and airways, modulating immune function, enhancing gastrointestinal microbiota, and restoring mitochondrial energy metabolism (Figure 1).

### 4.1 Regulation of inflammatory immunity

Gao et al. (2024) found that SLBZS significantly reduced the levels of TNF-α, IL-6, IL-8, and other inflammatory mediators in a COPD cell model induced by cigarette smoke extract (CSE), while also preventing cellular apoptosis. The process may pertain to the modulation of the Toll-like receptor (TLR) 9/nuclear factor kappa-B (NF-κB) pathway. A study by Ouyang and Xu (2019) investigated the impact of SLBZS on airway inflammation in young asthmatic rats, revealing that SLBZS diminished the infiltration of inflammatory cells in the lung tissue. The underlying mechanism may involve a reduction in IL-17 levels and an elevation in interleukin-10 (IL-10) and transforming growth factor-β1 (TGF-β1) levels. Li C. H. et al. (2024) established that SLBZS can reduce inflammatory infiltration in lung tissue, restore epithelial tissue shape, lower serum and alveolar lavage fluid concentrations of IL-17, and increase IL-10 levels. This may transpire via the regulation of the Th17/Treg equilibrium and the reestablishment of immunological function. Wang et al. (2020) found that SLBZS improves peak inspiratory flow (PIF), peak expiratory flow (PEF), and minute ventilation (MV) in rats with recurrent respiratory tract infection (RRTI), possibly associated with the modulation of serum immunoglobulin A (IgA), immunoglobulin G (IgG), immunoglobulin M (IgM), and other immune factors, along with a decrease in NF-κB p65 expression in the trachea. Furthermore, it inhibited the levels of inflammatory factors such as TNF-α, interleukin-1β (IL-1β) and IL-4 in serum. Deng et al. (2024) developed a rat model of idiopathic pulmonary fibrosis through intratracheal administration of bleomycin, discovering that modified SLBZS markedly diminished inflammatory infiltration and collagen proliferation in the lung tissue of rats, as well as reduced the expression of IL-1β and TNF-ɑ in lung tissue. The proposed mechanism of action may involve the modulation of the Nrf2/Keap1/NLRP3 signalling pathway, thereby delaying the pathological progression of idiopathic pulmonary fibrosis in rats through the regulation of pyroptosis.

### 4.2 Regulating the gut microbiota

Feng et al. (2020) found that SLBZS may reduce bacterial load in the bronchoalveolar lavage fluid of mice with pneumonia. It can reduce the levels of IL-1β, IL-6, TNF-α, interleukin-2 (IL-2), IL-8, interleukin-12 (IL-12), and IFN-γ in lung tissue homogenate, increase the concentrations of IL-10 and other inflammatory mediators, and improve lung injury in mice. The process may relate to the augmentation of species diversity and abundance of gut bacteria. Wu et al. (2022) established a juvenile Balb/c mouse model demonstrating intestinal flora dysbiosis and pulmonary inflammation via the injection of antibiotics alongside lipopolysaccharide. SLBZS was found to increase IL-10 levels in the bronchoalveolar lavage fluid of this juvenile mouse model, while simultaneously reducing levels of IFN-γ, TNF-α, and serum IgM. The procedure may entail improving the Shannon index of intestinal microbiota, adjusting the levels of Bacteroidetes, Firmicutes, and Proteobacteria, and mitigating the immune and inflammatory reactions in the lungs of juvenile rats. Ouyang et al. (2020) conducted a study that established a mouse model of asthma typified by intestinal flora dysbiosis induced by antibiotics, sensitisation, and aerosol challenge. The study indicated that SLBZS could reduce inflammatory infiltration in the lung tissue of model mice, possibly by increasing the relative abundance of key bacteria such Pseudoprevotella.

### 4.3 Regulation of energy metabolism

Inadequate skeletal muscle energy metabolism directly leads to respiratory failure in persons with COPD. Hu et al. (2020) established a mouse model of COPD with the application of cigarette smoke. SLBZS was discovered to protect mitochondrial energy metabolism in COPD mice by augmenting the expression of phosphorylated AMP-activated protein kinase (p-AMPK), peroxisome proliferator-activated receptor gamma coactivator-1α (PGC-1α), and mitochondrial fusion protein 2 (Mfn2), thereby maintaining normal skeletal muscle function in these mice. A separate study (Zhou et al., 2020) demonstrated that SLBZS enhances mitochondrial functions, including reactive oxygen species levels, mitochondrial ATP production, and membrane potential, mitigates oxidative damage, and decreases early apoptosis of cells via the PTEN induced putative kinase 1 (PINK1)/Parkin pathway-mediated mitophagy in a COPD myoblast model.

While the aforementioned research indicate that the mechanism of action of SLBZS in treating respiratory disorders may involve the reduction of inflammatory responses, the modulation of gut flora, and the regulation of energy metabolism, other limitations persist. 1) Experimental models exhibit variations. Contemporary research predominantly relies on animal models (e.g., bleomycin-induced lung fibrosis, COPD resulting from cigarette smoke exposure); nonetheless, notable discrepancies exist in the immune microenvironment and disease progression between these models and human conditions. The regulation mechanism of Th17/Treg equilibrium in murine asthma models may not completely replicate the heterogeneity of human asthma. 2) Disintegration of route analyses. Numerous studies indicate that pathways including NF-κB and NLRP3 are implicated in SLBZS; however, there is an absence of comprehensive study on the upstream and downstream elements of these pathways across the research. Is there cross-regulation between the TLR9/NF-κB pathway (Gao et al., 2024) and the NLRP3 pathway (Deng et al., 2024)? This division may obscure the fundamental objectives of SLBZS. The absence of validation for protein phosphorylation and the lack of TLR9 knockdown rescue trials complicate the exclusion of off-target effects. 3) Lack of flora specificity. Alterations in gut flora abundance have been noted to correlate with enhancements in lung inflammation in studies (Feng et al., 2020; Wu et al., 2022); however, these studies predominantly rely on correlation analyses, necessitating further validation of causality through faecal transplantation experiments or controlled antibiotic clearance studies of flora. Conversely, current research has solely documented alterations at the phylum level (e.g., *Mycobacterium* anisopliae, *Mycobacterium* thickum); however, distinct strains within the same phylum may exhibit divergent immunomodulatory effects, necessitating future functional validation at the strain level. 4) Clinical significance. Energy metabolism research often utilises acute damage models to assess mitochondrial function; however, skeletal muscle depletion in COPD patients is a protracted process. The sustainability of SLBZS in regulating AMPK/PGC-1α throughout the chronic progression of the disease requires more examination. Currently, research on energy metabolism has predominantly concentrated on skeletal muscle; however, the metabolic reprogramming of lung tissues, such as alveolar epithelial cells and fibroblasts, is equally vital in pulmonary fibrosis and asthma. Future studies should prioritise investigating whether SLBZS exerts a selective impact on mitochondria across various tissues.

## 5 Q-marker prediction analysis of SLBZS treatment for respiratory diseases

**FIGURE 2:**
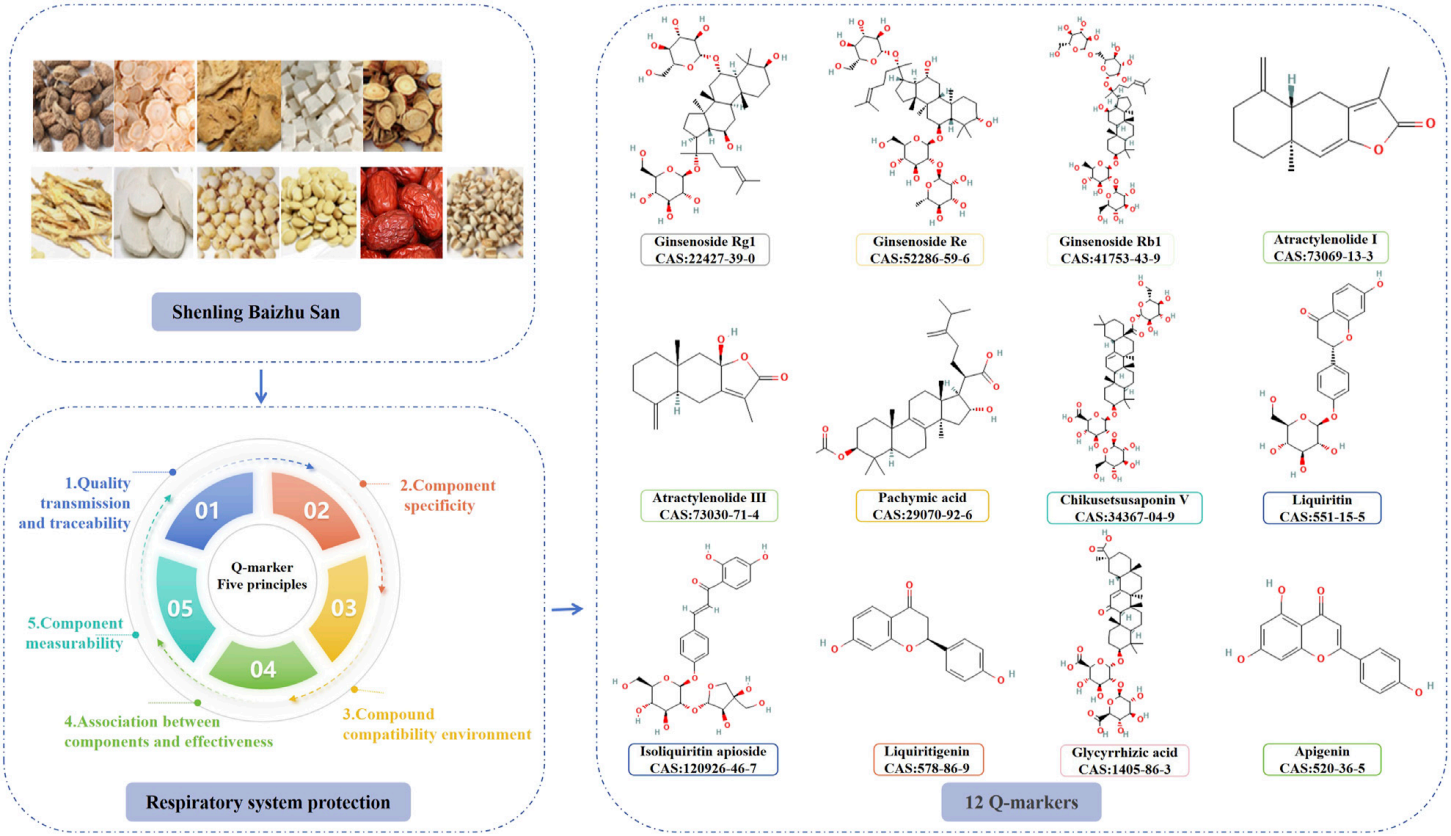
Steps in predicting Q-markers of SLBZS.

Substantial progress has been achieved in the clinical documentation and pharmacological investigation of SLBZS for respiratory illnesses; however, the active components necessitate further elucidation. Liu Changxiao, a member of the Chinese Academy of Engineering, presented the Q-marker for TCM botanical medications, a novel concept designed to enhance quality control and improve the quality of TCM formulae. It includes five principles: quality transmission and traceability, metabolites specificity, formula compatibility environment, association between metabolites and effectiveness, metabolites measurability (Liu, 2021). The five concepts of Q-markers were utilised to forecast prospective Q-markers for SLBZS in the management of respiratory disorders (Figure 2).

### 5.1 Q-marker prediction based on quality transmission and traceability

The metabolites are the essential foundation for the efficacy of TCM formulae Liquid chromatography, liquid chromatography-mass spectrometry, and more technologies can rapidly and accurately identify metabolites. In a study conducted by Ou et al. (2024), 83 metabolites were identified in the ethanol extract of SLBZS utilising UPLC-Q-TOF-MS/MS technology. These included 26 triterpenoid saponins, 15 flavonoid glycosides, eight flavonoids, seven triterpenoids, six alkaloids, five phospholipids, four fatty acids, four sesquiterpenoids, two fatty acid esters, two nucleotides, one nucleoside, one stilbene, one coumarin, and one phenanthrene. The metabolites of the aqueous extract of SLBZS were analysed using UPLC-Q-TOF-MS/MS technology, resulting in the identification of 104 metabolites, comprising 63 flavonoids, 14 organic acids, 13 terpenes, five coumarins, and nine other compounds (Li C. H. et al., 2024).

The blood constituents and metabolites of TCM botanical medications represent the primary active metabolites. Consequently, the examination of serum pharmacochemistry is essential for finding quality markers in TCM botanical drugs. Gao et al. (2024) used UPLC-Q-TOF-MS/MS technology to analyze SLBZS serum and identified a total of 108 metabolites, including 30 prototype components and 78 metabolites. Among them, 30 prototype components mainly included one alkaloid compound from Yiyiren. Four flavonoid glycosides from Gancao; There were 18 triterpene saponins, including 13 from Gancao, four from Renshen, and one from Baibiandou. There were three triterpene acids, two from Fuling and one from Jiegeng. Two flavonoids from Gancao; one sesquiterpenoid from Baizhu; one phospholipid compound from Baibiandou. The metabolic pathways of 78 metabolites mainly included glucuronidation, sulfation, methylation, hydroxylation, and acetylation. Li L. et al. (2024) using UPLC-Q-TOF to analyse serum containing SLBZS, identifying 11 prototype components and 17 metabolites. The eleven prototype components comprised eight flavonoids, one phenolic acid, one terpenoid, and one coumarin. The metabolic pathways of 17 metabolites primarily encompassed phase I metabolism, including methylation, reduction, and double reduction, as well as phase II metabolism, comprising glucuronidation and sulfation. Moreover, Xu et al. (2021) employed UHPLC-MS/MS technology, identifying the presence of panaxadiol, ginsenoside Rg1, atractylenolide I, atractylenolide III, pachymic acid, neferine, nuciferine, diosgenin, platycodin D, and isoliquiritigenin in the serum of rats administered SLBZS via gavage. The specifics of SLBZS in the bloodstream are presented in Table 3. Triterpene saponins, flavonoid glycosides, alkaloids, flavonoids, triterpenoids, and other metabolites are hypothesised to be the principal compounds by which SLBZS exerts its pharmacological effects.

**TABLE 3:** The metabolites of SLBZS.

No.	Compounds	Formula	Method	Refs
1	1-Hexadecanoyl-sn-glycero-3-phospho-(1′-myo-inositol)	C_25_H_49_O_12_P	Alcohol extraction, entering the blood	Gao et al. (2024)
2	(2E)​-​1-​(2,​4-​Dihydroxyphenyl)​-​3-​[4-​[[2-​O-​[2-​(1-​piperidinyl)​acetyl]​-​β-​D-​glucopyranosyl]​oxy]​phenyl]​-​2-​propen-​1-​one	C_28_H_33_NO_10_	Alcohol extraction	Ou et al. (2024)
3	(2E)-3-[4-[[6-O-D-Apio-β-D-furanosyl-2-O-[2-(1-piperidinyl)acetyl]-β-D-glucopyranosyl]oxy]phenyl]-1-(2,4-dihydroxyphenyl)-2-propen-1-one	C_33_H_41_NO_14_	Alcohol extraction	Ou et al. (2024)
4	(2E)-3-[4-[[6-O-D-Apio-β-D-furanosyl-2-O-[2-(1-piperidinyl)acetyl]-β-D-glucopyranosyl]oxy]phenyl]-1-(2,4-dihydroxyphenyl)-2-propen-1-one Isomer	C_33_H_41_NO_14_	Alcohol extraction	Ou et al. (2024)
5	(2S)​-​2,​3-​Dihydro-​7-​hydroxy-​2-​[4-​[[2-​O-​[2-​(1-​piperidinyl)​acetyl]​-​β-​D-​glucopyranosyl]​oxy]​phenyl]​-​4H-​1-​benzopyran-​4-​one	C_28_H_33_NO_10_	Alcohol extraction	Ou et al. (2024)
6	1-(9Z,12Z-Octadecadienoyl)-glycero-3-phospho-(1′-myo-inositol)	C_27_H_49_O_12_P	Alcohol extraction	Ou et al. (2024)
7	1-Linoleoyl-sn-glycero-3-phosphocholine	C_26_H_50_NO_7_P	Alcohol extraction	Ou et al. (2024)
8	1-Linoleoyl-sn-glycero-3-phosphoethanolamine	C_23_H_44_NO_7_P	Alcohol extraction	Ou et al. (2024)
9	1-O-Hexadecanoylhexitol	C_22_H_44_O_7_	Alcohol extraction	Ou et al. (2024)
10	1-Palmitoyl-sn-glycero-3-phosphoethanolamine	C_21_H_44_NO_7_P	Alcohol extraction	Ou et al. (2024)
11	22-acetoxyl-glycyrrhizin	C_44_H_64_O_18_	Alcohol extraction	Ou et al. (2024)
12	22-beta-Acetoxyglycyrrhetaldehyde	C_44_H_64_O_17_	Alcohol extraction, entering the blood	Gao et al. (2024)
13	22-beta-Acetoxyglycyrrhetaldehyde Isomer	C_44_H_64_O_17_	Enter the bloodstream	Gao et al. (2024)
14	22-dehydrouralsaponin C	C_42_H_64_O_15_	Alcohol extraction, entering the blood	Gao et al. (2024)
15	22β-acetoxyl-glycyrrhizin	C_44_H_64_O_18_	Enter the bloodstream	Gao et al. (2024)
16	2′-hydroxyisowighteone glucoside	C_25_H_26_O_6_	Water extraction	Li et al. (2024a)
17	2-O-Acetyl-Platyconic acid A	C_59_H_92_O_3_0	Alcohol extraction	Ou et al. (2024)
18	3-​(4-​Methoxyphenyl)​-​7-​[[2-​O-​[2-​(1-​piperidinyl)​acetyl]​-​β-​D-​glucopyranosyl]​oxy]​-​4H-​1-​benzopyran-​4-​one	C_29_H_33_NO_10_	Alcohol extraction	Ou et al. (2024)
19	3,4-Didehydroglabridin	C_20_H_18_O_4_	Water extraction	Li et al. (2024b)
20	3,5-dihydroxybenzoic acid	C_7_H_6_O_4_	Water extraction	Li et al. (2024a)
21	5-hydroxymethylfurfural	C_6_H_6_O_3_	Water extraction	Li et al. (2024a)
22	6-​Nitroveratric acid	C_9_H_9_NO_6_	Alcohol extraction	Ou et al. (2024)
23	6″-acetylvliquiritin	C_23_H_23_O_10_	Water extraction	Li et al. (2024b)
24	6-Nitroveratric acid	C_9_H_9_NO_6_	Enter the bloodstream	Gao et al. (2024)
25	7-hydroxy-6,4′-dimethoxyisoflavone	C_17_H_14_O_5_	Water extraction	Li et al. (2024a)
26	7-O-methyl-lupinisoflavone	C_21_H_20_O_6_	Water extraction	Li et al. (2024b)
27	Adenosine	C_10_H_13_N_5_O_4_	Water extraction	Li et al. (2024a)
28	Apigenin-7-O-glucoside	C_21_H_20_O_10_	Water extraction	Li et al. (2024b)
29	Apioglycyrrhizin	C_26_H_30_O_13_	Water extraction	Li et al. (2024a)
30	Araboglycyrrhizin	C_41_H_62_O_14_	Alcohol extraction, entering the blood	Gao et al. (2024)
31	Arginine	C_6_H_14_N_4_O_2_	Water extraction	Li et al. (2024b)
32	Armepavine	C_19_H_23_NO_3_	Alcohol extraction	Ou et al. (2024)
33	Artemisinic acid	C_15_H_22_O_2_	Water extraction	Li et al. (2024a)
34	Atractylenolide Ⅲ	C_15_H_20_O_3_	Alcohol extraction, water extraction, and entry into the blood	Gao et al. (2024), Xu et al. (2021)
35	Atractylenolide I	C_15_H_18_O_2_	Alcohol extraction, water extraction, and entry into the blood	Xu et al. (2021)
36	AtractylenolideⅡ Isomer	C_15_H_20_O_2_	Alcohol extraction	Ou et al. (2024)
37	Atractylon	C_15_H_20_O	Water extraction	Li et al. (2024b)
38	Azelaic acid	C_9_H_16_O_4_	Water extraction	Li et al. (2024a)
39	Batatasin I	C_17_H_16_O_4_	Alcohol extraction	Ou et al. (2024)
40	Batatasin III	C_15_H_16_O_3_	Alcohol extraction	Ou et al. (2024)
41	Betulinic acid	C_30_H_48_O_3_	Water extraction	Li et al. (2024b)
42	Biochanin A	C_16_H_16_O_4_	Water extraction	Li et al. (2024a)
43	Calycosin	C_16_H_12_O_5_	Water extraction	Li et al. (2024b)
44	cAMP Cyclic adenosine monophosphate	C_10_H_12_N_5_O_6_P	Alcohol extraction	Ou et al. (2024)
45	cAMP Isomer	C_10_H_12_N_5_O_6_P	Alcohol extraction	Ou et al. (2024)
46	Catechin	C_15_H_14_O_6_	Alcohol extraction, entering the blood	Ou et al. (2024)
47	Chikusetsusaponin V	C48H76O19	Alcohol extraction, entering the blood	Gao et al. (2024)
48	Chinensin	C_17_H_16_O_6_	Water extraction	Li et al. (2024a)
49	Chlorogenic acid A	C_25_H_24_O_12_	Water extraction	Li et al. (2024b)
50	Chlorogenic acid B	C_25_H_24_O_12_	Water extraction	Li et al. (2024a)
51	Corydine	C_20_H_23_NO_4_	Alcohol extraction	Ou et al. (2024)
52	Cynarin	C_25_H_24_O_12_	Water extraction	Li et al. (2024b)
53	Daidzein	C15H10O4	Water extraction, enters the blood	Li et al. (2024a)
54	Daidzin	C_22_H_22_O_10_	Water extraction	Li et al. (2024b)
55	Dehydrohispidol C	C_21_H_22_O_5_	Water extraction	Li et al. (2024a)
56	Dehydrohispidol D	C_22_H_24_O_5_	Water extraction	Li et al. (2024b)
57	Dehydropachymic acid	C_33_H_50_O_5_	Alcohol extraction	Ou et al. (2024)
58	Dehydrotumulosic acid	C31H48O4	Alcohol extraction, entering the blood	Gao et al. (2024)
59	Dihydroquercetin-7-O-rhamnoside	C_21_H_22_O_11_	Water extraction	Li et al. (2024a)
60	Diosgenin	C27H42O3	Enter the bloodstream	Xu et al. (2021)
61	Eleutherinolone	C_22_H_26_O_9_	Water extraction	Li et al. (2024b)
62	Epicatechin	C_15_H_14_O_6_	Alcohol extraction	Ou et al. (2024)
63	Ethyl palmitate	C_18_H_36_O_2_	Water extraction	Li et al. (2024a)
64	Formononetin	C_22_H_22_O_9_	Water extraction	Li et al. (2024b)
65	Formononetin	C_16_H_12_O_4_	Water extraction	Li et al. (2024a)
66	Fraxetin	C_27_H_30_O_14_	Water extraction	Li et al. (2024b)
67	Gancaonin E	C_25_H_28_O_6_	Alcohol extraction	Ou et al. (2024)
68	Gancaonin L	C_20_H_18_O_6_	Alcohol extraction	Ou et al. (2024)
69	Genistein	C_16_H_12_O_5_	Water extraction	Li et al. (2024a)
70	Genkwanin	C_16_H_12_O_5_	Water extraction	Li et al. (2024b)
71	Ginsenoside Rb1	C54H92O23	Alcohol extraction, entering the blood	Gao et al. (2024)
72	Ginsenoside Re	C48H82O18	Alcohol extraction, entering the blood	Gao et al. (2024)
73	Ginsenoside Re Isomer	C48H82O18	Enter the bloodstream	Gao et al. (2024)
74	Ginsenoside Rf	C_4_2H_72_O_14_	Alcohol extraction	Ou et al. (2024)
75	Ginsenoside Rg1	C42H72O14	Alcohol extraction, entering the blood	Gao et al. (2024), Xu et al. (2021)
76	Glabridin	C_20_H_20_O_4_	Water extraction	Li et al. (2024a)
77	Glabrolide	C_22_H_22_O_6_	Water extraction	Li et al. (2024b)
78	Glicophenone	C_22_H_22_O_6_	Water extraction	Li et al. (2024a)
79	Glisoliquiritigenin	C_22_H_22_O_6_	Water extraction	Li et al. (2024b)
80	Glycitin	C22H22O10	Enter the bloodstream	Li et al. (2024a)
81	Glycycoumarin	C21H20O6	Alcohol extraction, water extraction, and entry into the blood	Gao et al. (2024)
82	Glycyrol A	C_20_H_18_O_6_	Water extraction	Li et al. (2024b)
83	Glycyrol B	C_20_H_16_O_6_	Water extraction	Li et al. (2024a)
84	Glycyronin E	C_25_H_28_O_6_	Water extraction	Li et al. (2024b)
85	Glycyronin H	C_25_H_24_O_6_	Water extraction	Li et al. (2024a)
86	Glycyronin L	C_20_H_18_O_6_	Water extraction	Li et al. (2024b)
87	Glycyrrhetolide	C_30_H_44_O_4_	Water extraction	Li et al. (2024a)
88	Glycyrrhizic acid	C42H62O16	Alcohol extraction, entering the blood	Gao et al. (2024)
89	Glycyrrhizic acid G2	C_42_H_62_O_17_	Water extraction	Li et al. (2024b)
90	Glycyrrhizic acid Isomer	C42H62O16	Alcohol extraction, entering the blood	Gao et al. (2024)
91	Glyyunnansapogenin C	C_30_H_44_O_4_	Water extraction	Li et al. (2024a)
92	Glyyunnansapogenin E	C30H46O5	Water extraction, enters the blood	Li et al. (2024b)
93	Hedysarumscoparium coumestrol B	C_16_H_10_O_6_	Water extraction	Li et al. (2024a)
94	Hispidin A	C_25_H_26_O_6_	Water extraction	Li et al. (2024b)
95	Hispidin C	C_21_H_24_O_5_	Water extraction	Li et al. (2024a)
96	Hispidin D	C_22_H_26_O_5_	Water extraction	Li et al. (2024b)
97	Homoplantaginin	C_16_H_14_O_5_	Water extraction	Li et al. (2024a)
98	Hordenine	C_16_H_12_O_4_	Water extraction	Li et al. (2024b)
99	Isoapioglycyrrhizin	C_26_H_30_O_13_	Water extraction	Li et al. (2024a)
100	Isococlaurine	C_17_H_19_NO_3_	Alcohol extraction	Ou et al. (2024)
101	Isofraxidin	C_27_H_30_O_14_	Water extraction	Li et al. (2024b)
102	Isoliquiritigenin	C_15_H_12_O_4_	Water extraction, enters the blood	Xu et al. (2021)
103	Isoliquiritin	C_21_H_22_O_9_	Alcohol extraction, water extraction, and entry into the blood	Gao et al. (2024)
104	Isoliquiritin apioside	C_26_H_30_O_13_	Alcohol extraction, entering the blood	Gao et al. (2024)
105	Isolupalbigenin	C_25_H_26_O_5_	Water extraction	Li et al. (2024a)
106	Isorhamnetin-3-O-neohesperidoside	C_28_H_32_O_16_	Water extraction	Li et al. (2024b)
107	Isoschaftoside	C_26_H_28_O_14_	Water extraction, enters the blood	Li et al. (2024a)
108	Isovitexin	C_21_H_20_O_12_	Water extraction	Li et al. (2024b)
109	Kaempferol-3-O-robinobioside	C_27_H_30_O_16_	Water extraction	Li et al. (2024a)
110	Kaempferol-7-O-glucoside	C_21_H_20_O_11_	Water extraction	Li et al. (2024b)
111	Leucine	C_6_H_13_NO_2_	Water extraction	Li et al. (2024a)
112	Licocoumarone	C_20_H_20_O_5_	Water extraction, enters the blood	Li et al. (2024b)
113	Licoflavonol	C_20_H_18_O_6_	Alcohol extraction	Ou et al. (2024)
114	Licoisoflavone A	C_20_H_18_O_6_	Alcohol extraction	Ou et al. (2024)
115	Licorice chalcone B	C_16_H_14_O_5_	Water extraction	Li et al. (2024b)
116	Licorice chalcone C	C_21_H_22_O_4_	Water extraction	Li et al. (2024a)
117	Licorice chalcone D	C_21_H_22_O_5_	Water extraction	Li et al. (2024b)
118	Licorice chalcone E	C_21_H_22_O_4_	Water extraction	Li et al. (2024a)
119	Licorice flavonoid C	C_20_H_18_O_5_	Water extraction	Li et al. (2024b)
120	Licoricesaponin A3	C_48_H_72_O_21_	Alcohol extraction, entering the blood	Gao et al. (2024)
121	Licoricesaponin B2	C_42_H_64_O_15_	Alcohol extraction, entering the blood	Gao et al. (2024)
122	Licoricesaponin C2	C_42_H_62_O_15_	Alcohol extraction, entering the blood	Gao et al. (2024)
123	Licoricesaponin C2 Isomer	C_42_H_62_O_15_	Alcohol extraction	Ou et al. (2024)
124	Licoricesaponin E2	C_42_H_60_O_16_	Alcohol extraction, entering the blood	Gao et al. (2024)
125	Licoricesaponin G2	C_42_H_62_O_17_	Alcohol extraction, entering the blood	Gao et al. (2024)
126	Licoriphenone	C_21_H_24_O_6_	Water extraction, enters the blood	Li et al. (2024a)
127	Lignoceric acid	C_24_H_48_O_2_	Water extraction	Li et al. (2024b)
128	Linolenic acid	C_18_H_30_O_2_	Water extraction	Li et al. (2024a)
129	Liquiritigenin	C_15_H_12_O_4_	Alcohol extraction, water extraction, and entry into the blood	Gao et al. (2024), Li et al. (2024a)
130	Liquiritigenol	C_21_H_18_O_6_	Water extraction	Li et al. (2024b)
131	Liquiritin	C_21_H_22_O_9_	Alcohol extraction, water extraction, and entry into the blood	Gao et al. (2024); Li et al. (2024a)
132	Liquiritin apioside	C_26_H_30_O_13_	Enter the bloodstream	Gao et al. (2024)
133	Liquiritinapioside	C_26_H_30_O_13_	Alcohol extraction	Ou et al. (2024)
134	Lotusine	C_19_H_24_NO_3_^+^	Alcohol extraction	Ou et al. (2024)
135	Malic acid	C4H6O5	Water extraction	Li et al. (2024a)
136	Malonyl + Isoliquiritin apioside	C_29_H_32_O_16_	Alcohol extraction	Ou et al. (2024)
137	Malonyl + Liquiritinapioside	C_29_H_32_O_16_	Alcohol extraction	Ou et al. (2024)
138	Mannide monooleate	C_24_H_44_O_7_	Alcohol extraction	Ou et al. (2024)
139	Medicarpin	C_16_H_14_O_4_	Water extraction	Li et al. (2024b)
140	Naringenin	C_15_H_12_O_5_	Water extraction	Li et al. (2024b)
141	Neferine	C_38_H_44_N_2_O_6_	Alcohol extraction, entering the blood	Xu et al. (2021)
142	Neoisoliquiritin	C_21_H_22_O_9_	Alcohol extraction	Ou et al. (2024)
143	Nuciferine	C_19_H_21_NO_2_	Enter the bloodstream	Xu et al. (2021)
144	Oleanolic acid	C_30_H_48_O_3_	Alcohol extraction	Ou et al. (2024)
145	Orientin	C_21_H_20_O_11_	Water extraction, enters the blood	Li et al. (2024b)
146	Pachymic acid	C_33_H_52_O_5_	Alcohol extraction, entering the blood	Xu et al. (2021)
147	Pachymic acid B	C_30_H_44_O_5_	Water extraction	Li et al. (2024b)
148	Palmitic acid	C_16_H_32_O_2_	Alcohol extraction	Ou et al. (2024)
149	Panaxadiol	C_30_H_52_O_3_	Enter the bloodstream	Xu et al. (2021)
150	Phenylalanine	C_9_H_11_NO_2_	Water extraction	Li et al. (2024b)
151	P-hydroxycinnamic acid	C_9_H_8_O_3_	Water extraction	Li et al. (2024a)
152	Pinellic acid	C_18_H_34_O_5_	Alcohol extraction	Ou et al. (2024)
153	Platycodin A	C_59_H_94_O_29_	Alcohol extraction	Ou et al. (2024)
154	Platycodin C	C_59_H_94_O_29_	Alcohol extraction	Ou et al. (2024)
155	Platycodin D	C_57_H_92_O_28_	Alcohol extraction, entering the blood	Xu et al. (2021)
156	Platycodin D2	C_63_H_102_O_33_	Alcohol extraction	Ou et al. (2024)
157	Platycodin E	C_62_H_116_O_43_	Alcohol extraction	Ou et al. (2024)
158	Platycodins K	C_59_H_92_O_30_	Alcohol extraction	Ou et al. (2024)
159	Platycogenic acid A	C_57_H_90_O_29_	Alcohol extraction, entering the blood	Gao et al. (2024)
160	Polyporenic acid C	C_31_H_46_O_4_	Alcohol extraction	Ou et al. (2024)
161	Poricoic acid C	C_31_H_46_O_4_	Water extraction	Li et al. (2024b)
162	Protocatechuic acid	C_7_H_6_O_4_	Water extraction, enters the blood	Li et al. (2024a)
163	Quercitrin	C_21_H_20_O_11_	Alcohol extraction	Ou et al. (2024)
164	Rhamnose	C_6_H_14_O_6_	Water extraction	Li et al. (2024b)
165	Salicylic acid	C_7_H_6_O_3_	Water extraction	Li et al. (2024a)
166	Schaftoside	C_27_H_30_O_15_	Water extraction	Li et al. (2024b)
167	Schaftoside	C_26_H_28_O_14_	Water extraction	Li et al. (2024a)
168	Semiglycyrol B	C_20_H_16_O_6_	Water extraction	Li et al. (2024b)
169	Tetrahydroxymethoxychalcone	C_16_H_14_O_6_	Water extraction	Li et al. (2024a)
170	Tumulosic acid	C_31_H_50_O_4_	Alcohol extraction, entering the blood	Gao et al. (2024)
171	Tyrosine	C_9_H_11_NO_3_	Water extraction	Li et al. (2024b)
172	Uralensis saponin N	C_24_H_62_O_17_	Water extraction	Li et al. (2024a)
173	Uralol	C_20_H_18_O_7_	Water extraction	Li et al. (2024b)
174	Uralsaponin B	C_42_H_62_O_16_	Alcohol extraction, entering the blood	Gao et al. (2024)
175	Ursolic Acid	C_30_H_48_O_3_	Alcohol extraction	Ou et al. (2024)
176	Vanillic acid	C_8_H_8_O_4_	Water extraction	Li et al. (2024b)
177	Vernolic acid	C_18_H_32_O_3_	Alcohol extraction	Ou et al. (2024)
178	Wighteone	C_20_H_18_O_5_	Water extraction	Li et al. (2024a)
179	Xenognosin B	C_16_H_12_O_5_	Water extraction	Li et al. (2024b)

### 5.2 Q-marker prediction based on metabolites specificity

Renshen is the desiccated root and rhizome of *Panax ginseng* C. A. Mey. It mostly consists of flavonoids, polysaccharides, saponins, and various other compounds. Ginsenosides are considered the principal active component among them (Ratan et al., 2020). The principal metabolites of Renshen include ginsenoside Rb1, ginsenoside Re, and ginsenoside Rg1 (Han et al., 2018).

Baizhu is the desiccated rhizome of *Atractylodes macrocephala* Koidz. It mostly comprises sesquiterpenes, triterpenes, polysaccharides, and other constituents (Zhu L. X. et al., 2018). Atractylenolide I and atractylenolide III serve as the distinctive metabolites of Baizhu (Xie et al., 2023).

Fuling is the desiccated sclerotia of *Poria cocos* (Schw.) Wolf, mostly consisting of triterpenoids, polysaccharides, sterols, diterpenoids, and other chemicals. Triterpenoids are widely regarded as the distinctive metabolites of Fuling (Zhu L. et al., 2018; Zhu et al., 2020).

Shanyao is the desiccated rhizome of *Dioscorea opposita* Thunb. The primary active constituents of Shanyao include phenolic acids, flavonoids, and polysaccharides. Batatasins and dioscin serve as the distinctive metabolites of Shanyao (Liu et al., 2024; Chen et al., 2020).

Lianzi is the desiccated and mature seed of *Nelumbo nucifera* Gaertn. Lianzi primarily comprises alkaloids, polyphenols, triterpene saponins, and other constituents. Neferine and nuciferine serve as the distinctive metabolites of lotus seed (Pei, 2021).

Yiyiren is the desiccated and mature seed of *Coix lacryma-jobi* L. var. *Mayuen* (Roman.) Stapf, primarily composed of esters, fatty acids, polysaccharides, and phenols (Pan et al., 2023). Fatty acids and their lipids constitute the active components of Yiyiren, while Coixenolide serves as a distinctive metabolite of Yiyiren (Lu et al., 2022).

Baibiandou is the desiccated and fully developed seed of the leguminous species *Dolichos lablab* L. It mostly comprises polysaccharides, saponins, alkaloids, amino acids, and various other chemical constituents. Total saponins of white lentil and pekolic acid serve as distinctive metabolites of Baibiandou (Li, 2018; Han, 2021).

Jiegeng is the desiccated root of *Platycodon grandiflorum* (Jacq.) A. DC., mostly comprising saponins, flavonoids, phenolic acids, and various other chemical constituents. Platycodin serves as a distinctive metabolite of Jiegeng (Zhang et al., 2022).

Gancao refers to the desiccated roots and rhizome of *Glycyrrhiza uralensis* Fisch. Gancao primarily comprises triterpene saponins, flavonoids, coumarin, polysaccharides, and various other chemical constituents. Glycyrrhizic acid and celiose-glycyrrhizin serve as distinctive metabolites of Gancao (Cheng et al., 2021; Shang et al., 2022).

Sharen is the desiccated and mature fruit of *Amomum villosum* Lour., *Amomum villosum* Lour. var. *xanthioides* T. L. Wu et Senjen, or *Amomum longiligulare* T. L. Wu, primarily comprising various chemical constituents, including volatile oil, which is typically regarded as the active component of amomum kernel, with bornyl acetate serving as a distinctive metabolite of Sharen (Feng et al., 2024).

Dazao is the desiccated and mature fruit of *Ziziphus jujuba* Mall. The composition mostly includes sugars, triterpenoids, phenolic acids, and other constituents, with ursolic acid and oleanolic acid serving as the distinctive metabolites of Dazao (Lu et al., 2021).

### 5.3 Q-marker prediction based on the formula compatibility environment

This TCM formula consists of Renshen, Baizhu, Fuling, Shanyao, Lianzi, Baibiandou, Sharen, Jiegeng, Gancao, and Dazao in the proportions of 15:15:15:15:9:9:12:6:6:10:7.5. Renshen has the ability to enhance qi levels in the body, particularly in the spleen and lungs. Baizhu exhibits properties of moisture desiccation, spleen energising, deficiency replenishment, and qi enhancement, whereas Fuling is distinguished by its capacity to alleviate water retention, promote moisture, and stimulate the spleen. The aforementioned three herbs are crucial in SLBZS. Shanyao enhances spleen and stomach function, while Lianzi also promotes spleen and stomach vitality, hence augmenting the effects of spleen and qi invigorating. Both Baibiandou and Yiyiren can enhance the efficacy of spleen fortification and dampness reduction. Sharen is capable of both dehumidifying and regulating the air machine. Jiegeng facilitates the enhancement of lung qi. Gancao nourishes qi, while Dazao nourishes the spleen and stomach, serving as adjunctive medications. The amalgamation of these TCM plant medications aims to enhance qi, stimulate the spleen, strengthen the lung, and promote the expulsion of moisture.

In clinical practice, individual TCM herbal remedies are often amalgamated with supplementary botanical medicines to generate a compound. The efficacy and potential pharmacodynamic qualities of TCM botanical drugs depend on the formulation or dosage of the plant. Consequently, it is imperative to forecast Q-markers associated with lung function preservation by integrating TCM within a composite context. A study (Zhang, 2019) demonstrated that the concentrations of ginsenosides Rg1, Re, Rf, Rb1, Rc, Rb2, and Rd elevated, whereas the concentration of atractone diminished after the Renshen-Baizhu compatibility. Another study (Li et al., 2014) indicated a significant increase in the concentrations of ginsenosides Rg1, Re, Rf, Rd, and atractylenolide I in Renshen-Baizhu compatibility. The levels of ginsenosides Rb3 and F1 were significantly elevated in the Renshen-Shanyao compatibility study, whereas the concentrations of 16 ginsenosides, including Rg1, Re, Rf, Rb1, Rg2, Rc, Rb2, Rd, F2, Rg3, protopanaxriol, CK, Rh2, and the total sugar content, were markedly reduced (Zhao, 2015). Yue et al. (2018) analysed the constituents of volatile oil following the compatibility of Baizhu and Fuling, discovering the presence of new components, including 14 substances such as 5-methylfuran aldehyde, glycyrene, terpene olefin, carpinene, and linolenic acid. A subsequent investigation (Wang et al., 2021) demonstrated a considerable rise in the concentrations of platycodin D, glycyrrhizic acid, and liquiritin when Jiegeng-Gancao was combined. The synergistic interaction between active components and certain herbs often accounts for the efficacy and therapeutic effects of TCM formulae. A single plant exhibits various pharmacological metabolites and mechanisms of action in a complex environment, resulting in unique therapeutic advantages.

### 5.4 Q-marker prediction based on theassociation between metabolites and effectiveness

The metabolites of a drug dictate its pharmacological impact and provide the essential element of Q-marker, which is vital for monitoring prescription quality. Studies suggest that the metabolites of SLBZS in treating respiratory disorders may be associated with the chemical components absorbed in the serum of Renshen, Baizhu, Fuling, Baibiandou, and Gancao. The mechanism of action of the active constituents of SLBZS is depicted in Table 4.

**TABLE 4:** Mechanism of action of SLBZS active ingredients.

No.	Active ingredient	Model and dosage of administration	The medication time	Control drugs	Effect	Mechanism	Refs
1	Apigenin	Model of lung injury: mouse (20, 50 mg/kg), Primary Splenocytes	7 days		Anti-inflammatory, anti-oxidative stress	Downregulated MDA, IL-6 and TNF-α, upregulated SOD, GSH-PX, CAT, IL-2, CD4^+^ and CD8^+^	Liu et al. (2018)
2	Apigenin	Model of asthma: mouse (10, 20 mg/kg), HBE (20 μM)	6 weeks; 16 h	Dexamethasone; selonsertib	Anti-inflammation, anti-apoptosis, anti-oxidative stress	Modulated ROS-ASK1-MAPK pathway, downregulated IL-5, IL-4, IL-13, IL-17, TNF-α, IFN-γ, T-bet, Gata3, RORγ-t, p-ASK1/ASK1, p-JNK/JNK, p-p38/p38, p-ERK/ERK, cyto-chrome c, Bax, caspase-3, upregulated Foxp3, Bcl-2	Yu et al. (2023)
3	Apigenin	Model of COPD: WI-38 cells (10, 20, 40 μM)	24 h	Resveratrol	Anti-oxidative stress, anti-aging	Modulated SIRT1-NAD + -CD38 Axis, downregulated SA-β-gal, ac-p53, p21, p16 and CD38, upregulated p-Rb, cyclin D1, SIRT1, NAD+, NAD+/NADH.	Li et al. (2021a)
4	Atractylolide I	Model of lung injury: mouse (5, 10, 20 mg/kg)	5 h	VGX-1027	Anti-inflammatory	Inhibition of TLR4/NF-κB pathway, downregulate TNF-α, IL-6, IL-1β, IL-13 and MIF, and upregulate IL-10	Zhang et al. (2015)
5	Atractylolide I	Model of recurrent respiratory tract infection: rat (3.33, 13.32 mg/kg)	6 weeks	Amoxicillin and clavulanate potassium	Anti-inflammatory, anti-oxidative stress	Inhibition of PI3K/Akt/mTOR pathway, downregulated IL-6, TNF-α, MDA, p-PI3K, p-Akt, p-mTOR, and upregulated SOD.	Wang et al. (2024c)
6	Atractylolide III	Model of lung injury: mouse (2, 8 mg/kg)	24 h		Anti-inflammatory, anti-apoptosis	Upregulation of Bcl-2, downregulation of IL-1β, TNF-α, IL-6, Bax, caspase-3, VNN1 and FoxO1	Fu et al. (2021)
7	Atractylolide III	Model of silicosis: mouse (30 mg/kg)	28 days and 56 days		Activating autophagy, anti-apoptosis	Activation of EGFR-mTOR pathway, downregulated LC3-II/I, Beclin1, p62, caspase 9, caspase 3, Col-1 and α-SMA, and upregulated LAMP2, p-EGFR, p-PI3K and p-Akt	Tan et al. (2023)
8	Atractylolide III	Model of asthma: mouse (25 mg/kg); 16HBE (100 ng/μL)	10 days; 48 h		Anti-apoptosis	Inhibit the activation of NLRP3 inflammasome, restore Th1/Th2 balance, downregulate caspase-1, ASC, NLRP3, IL-4, IL-13, and upregulate IL-12, γ-IFN.	Zhu et al. (2020)
9	Atractylolide III	Model of asthma: mouse (0.1, 1, 10 mg/kg)	7 days	dexamethasone	Anti-inflammatory, anti-oxidative stress	Inhibition of STAT3, upregulation of IFN-γ, IL-10, IL-12, GSH, SOD, CAT, downregulation of IL-4, IL-5, IL-13, ROS, MDA, LDH.	Zhang et al. (2021)
10	Atractylolide III	Model of lung injury: rat (0.6, 1.2, 2.4 mg/kg)	28 days		Anti-oxidative stress	Activation of Nrf2/NQO1/HO-1 pathway, downregulate Caspase-3, Caspase-9, TGF-β, α-SMA, IL-6, iNOS, TNF-α, MDA, LDH, and upregulate IL-10, SOD, GSH, Nrf2, NQO1 and HO-1	Huai and Ding. (2020)
11	Chikusetsusaponin V	Model of lung injury: mouse (5, 10, 20 mg/kg)	4 days		Anti-inflammatory	Inhibition of NF-κB pathway, downregulation of TNF-α, IL-1β, IL-6, p-p65, p-I-κB, upregulation of LXRα	Su et al. (2019)
12	Ginsenoside Rb1	Model of lung injury: mouse (10, 20 mg/kg), RAW 264.7 cells (10, 20 μg/mL)	24 days; 1 h		Anti-inflammatory	Inhibition of NF-κB and MAPK pathways, downregulation of IL-1β, IL-6, TNF-α, TLR2, p-p65, p-ERK, p-JNK.	Shaukat et al. (2019)
13	Ginsenoside Rb1	Model of asthma: mouse (10, 20 mg/kg)	3 days	Dexamethasone	Anti-inflammatory	Downregulated Th1/Th2, IL4, GATA3, upregulated IFN-γ, T-bet.	Chen et al. (2015)
14	Ginsenoside Re	Model of Ⅱ/R lung injury:rat (8, 16 mg/kg)	5 days		Anti-inflammatory, anti-oxidative	Downregulated IL-6, TNF-α, IL-10, MDA, and upregulated SOD	Fang et al. (2014)
15	Ginsenosides Rg1	Model of lung injury: mouse (30 mg/kg), MLE-12 cells (25 μg/mL)	24 h; 1 h		Activating autophagy, Anti-apoptosis	Downregulated p-p65, upregulated Nrf2	Ji et al. (2021)
16	Ginsenosides Rg1	Model of pulmonary inflammation: mouse (10, 20 mg/kg), A549 cells (12.5, 25, 50 μM)			Anti-inflammatory, anti-endoplasmic reticulum stress	Downregulated TNF-α, IL-1β, IL-6, p-p65, iNOS, CHOP, GRP78, IRE1α and ATF6, upregulated SIRT1	Wang et al. (2019)
17	Ginsenosides Rg1	Model of lung injury: mouse (40, 200 mg/kg)	7 h		Anti-inflammatory, regulation of M2 macrophage infiltration, anti-apoptosis	Downregulation of NF-κB, caspase-3, TNF-α, IL-1β and IL-6	Bao et al. (2015)
18	Ginsenosides Rg1	Model of COPD: rat (5, 10, 20 mg/kg), HBE cells (40 μM)	12 weeks; 48 h		Anti-Pulmonary Epithelial-Mesenchymal Transition	Inhibition of TGF-β1/Smad pathway, downregulation of α-SMA, TGF-β1, TGF-βR1, phospho-Smad2, phospho-Smad3, upregulation of E-cad	Guan et al. (2017)
19	Glycyrrhizic acid	Model of lung injury: rat (15 mg/kg)	3 months		Anti-inflammatory, anti-oxidative stress, anti-pulmonary fibrosis	Downregulated MDA, TNF-a, TGB-β, IL-1β, MTC stain, αSMA, CD68, upregulated GSH, TAC.	Elsherbini et al. (2021)
20	Glycyrrhizic acid	Model of lung injury: mouse (200 mg/kg), RAW264.7 cells (100 μg/mL)	25 h; 25 h		Anti-inflammatory, activates autophagy	Modulated PI3K/AKT/mTOR pathway, downregulated TNF-α, IL-1β, HMGB1, P62, p-PI3K/PI3K, p-AKT/AKT, p-mTOR/mTOR, upregulated LC3-II/LC3-I, Beclin-1	Qu et al. (2019)
21	Glycyrrhizic acid	Model of lung injury: rat (25, 50 mg/kg)	24 h		Anti-inflammatory, anti-apoptosis, anti-oxidative stress	Inhibition of NF-κB and MAPK pathways, downregulated TNF-α, IL-1β, IL-6, NO, iNOS, MDA, 8-OHdG, NT, caspase-3, P-i-κB-α, DNA binding activity of NF-κB, p-JNK/JNK, p-p38/p38, Upregulated SOD, Bcl-2/Bax and IκB-α	Zhao et al. (2016)
22	Glycyrrhizic acid	Model of lung injury: mouse (10, 20, 40 mg/kg)	5 days	dexamethasone	Anti-inflammatory, immune regulation	Downregulated IL-4, IL-5 and IL-13, upregulated IFN-γ, Tregs and Foxp3	Ma et al. (2013)
23	Liquiritigenin	Model of lung injury: *S. aureus* strain 8325–4 (4, 8, 16, 32 mg/mL)	30 min		Anti-staphylococcus aureus	Inhibition of alpha-hemolysin	Dai et al. (2013)
24	Liquiritigenin	Model of pulmonary fibrosis: mouse (25, 50, 100 mg/kg), Primary mouse lung fibroblasts (3, 10, 30, 100 μM)	14 days; 2 h		Anti-fibrosis, anti-oxidative stress	Activation of SIRT1/Nrf2 pathway, downregulated collagen I, α-SMA, GSH, SOD and ROS, upregulated MDA, CAT, HO-1, NQO-1 and SIRT1	Hua and Ren. (2024)
25	Liquiritin	Model of lung injury: Zebrafish (25, 50, 100 μM), mouse (40, 80 mg/kg), RAW264.7 (100, 200,300 μM)	0–80 h; 7 days; 2 h	dexamethasone	Anti-inflammatory	Inhibition of JNK/Nur77/c-Jun pathway, downregulation of TNF-α, IL-6, p-JNK, p-Nur77, p-c-Jun, upregulation of Nur77	Zhou et al. (2023)
26	Liquiritin	Model of lung injury: mouse (25, 50, 100 mg/kg), THP-1 cells (25, 50, 100 μM)	12 h; 1 h		Anti-inflammatory	Inhibition of NF-κB pathway, downregulated TNF-α, IL-6, IL-1β, TRPV1, TRPA1, p-p65/p65, p-I-κBα/I-κBα	Liu et al. (2020)
27	Liquiritin	Model of lung injury mouse (20, 50 mg/kg), MLE-2 cells (50 μM)	7 days; 3 h		Anti-inflammation, anti-iron death	Downregulated MDA, Fe+, TNF-α, Il-6, Hmgb1, Hif-1α, HO-1, Ptgs2 and Acsl4, upregulated GSH and Gpx4	Zhongyin et al. (2022)
28	Pachymaric acid	Model of pulmonary fibrosis: rat (10, 20, 40 mg/kg)	14 days		Inhibit endoplasmic reticulum stress, anti-oxidative stress, improve mitochondrial function	Downregulated Hyp, TGF-β1, collagen I, α-SMA, fibronectin, MDA, ROS, GRP78, CHOP, Caspase 9 and ATF4, upregulated SOD, CAT and ATP.	Li et al. (2023)
29	Pachymaric acid	Model of lung injury: mouse (25, 50, 100 mg/kg)	28 days	Prednisone acetate tablets	Anti-inflammatory, anti-oxidative stress	Downregulated IL-6, TNF-α, HYD, MDA, NLRP3, ASC, IL-1β, P20, TXNIP, upregulated IL-10, SOD, GSH-Px	Wang et al. (2023)
30	Pachymaric acid	Model of Pneumonia: rat (10, 20 mg/kg)	3 days		Anti-inflammatory, anti-apoptosis	Inhibition of NF-κB and MAPK pathways, downregulated IL-6, IL-1β, TNF-α, MCP-1, BAX, p-p65/p65, p-p38/p38, p-ERK1/2/ERK1/2, upregulated Bcl-2	Gui et al. (2021)

#### 5.4.1 Renshen

Ginsenosides, particularly Rg1, Re, and Rb1, are the unique metabolites of Renshen and serve as the bioactive components of SLBZS, demonstrating considerable therapeutic effectiveness against acute lung injury, pneumonia, asthma, and various respiratory disorders. Ji et al. (2021) established a cellular model of acute lung injury by subjecting lung epithelial cells to lipopolysaccharide (LPS) exposure. They subsequently discovered that ginsenoside Rg1 may inhibit cell death by augmenting autophagy and Nrf2 expression. A study by Wang et al. (2019) indicates that ginsenoside Rg1 can alleviate inflammation and lung injury caused by sepsis. This protective effect may be attributed to the decrease of inflammatory factors TNF-α, IL-1β, and IL-6 in lung tissue, together with the alleviation of endoplasmic reticulum stress. Furthermore, ginsenoside Rg1 can suppress lung epithelial-mesenchymal transition via modulating the infiltration of M2 macrophages in lung tissues (Bao et al., 2015; Guan et al., 2017). In a rat model of intestinal ischemia/reperfusion (I/R) lung injury, Fang et al. (2014) demonstrated that ginsenoside Re increased serum levels of TNF-α, IL-6, IL-10, superoxide dismutase (SOD), and malonaldehyde (MDA), showcasing its antioxidative and anti-inflammatory effects. Shaukat et al. (2019) assessed the anti-inflammatory properties of ginsenoside Rb1 in mice with acute lung injury, discovering its regulation of NF-κB and mitogen-activated protein kinase (MAPK) signalling pathways via TLR2, therefore preserving lung function in the subjects. Furthermore, ginsenoside Rb1 may exert an anti-asthmatic effect by modulating the Th1/Th2 balance (Chen et al., 2015).

#### 5.4.2 Baizhu

Atractylolide I and atractylolide III are the principal metabolites of Baizhu. Research has shown that atractylolide I and atractylolide III provide advantageous effects on lung damage, asthma, pulmonary fibrosis, and other ailments. In an *in vitro* investigation, Fu et al. (2021) demonstrated that atractonolide III could ameliorate lung injury caused by sepsis, potentially through the inhibition of Forkhead box protein O1 (FoxO1) and Vanin 1 (VNN1) protein expression, thereby decreasing lung tissue inflammation and suppressing cellular apoptosis. Atractylenolide III was demonstrated in a separate investigation to enhance autophagy failure, subsequently reducing apoptosis and alleviating lung fibrosis in silicotic mice (Tan et al., 2023). Atractylodes III can suppress the activation of the nucleotide-binding oligomerisation domain, NLRP3 inflammasome, restore Th1/Th2 balance in an asthma animal model, prevent death of bronchial epithelial cells, and demonstrate significant anti-asthma efficacy (Zhu et al., 2020). Moreover, research indicates that atractylenolide III can reduce oxidative stress in lung tissue and mitigate the symptoms of pulmonary fibrosis, asthma, and other conditions (Zhang et al., 2021; Huai and Ding, 2020). Atractylenolide I mitigates lung function impairment and reduces the inflammatory response in mice with acute lung injury via inhibiting TLR4 expression and NF-κB activation (Zhang et al., 2015). Atractylenolide I can also modulate the Phosphoinositide 3-kinase (PI3K)/protein kinase B (Akt)/mammalian target of rapamycin (mTOR) signalling pathway to enhance lung function in a rat model of recurrent respiratory infections (Wang et al., 2024b).

#### 5.4.3 Fuling

Pachymaric acid shown an optimum protective effect on lung function in the study of anti-pulmonary fibrosis and anti-pneumonia. A study (Li et al., 2023) demonstrated that pachymic acid can mitigate the symptoms of pulmonary oedema and pulmonary fibrosis in rats, potentially via decreasing endoplasmic reticulum stress and enhancing mitochondrial activity. Pachymic acid may also mitigate lung fibrosis by diminishing inflammatory responses and oxidative stress (Wang et al., 2023). Pachymic acid may alleviate pneumonia symptoms in rats by reducing lung inflammation and avoiding cell death, potentially through the modulation of the NF-κB and MAPK signalling pathways (Gui et al., 2021).

#### 5.4.4 Baibiandou

Chikusetsusaponin V, derived from Baibiandou, has shown effectiveness in alleviating acute lung injury. Su et al. (2019) developed a mouse model of acute lung injury utilising lipopolysaccharide and found that chikusetsusaponin V ameliorated lung pathological damage by reducing the concentrations of TNF-α, IL-1β, IL-6, and other inflammatory mediators in alveolar lavage fluid, while also inhibiting the activation of the NF-κB signalling pathway.

#### 5.4.5 Gancao

Glycyrrhizin, isorhamnetin, glycyrrhizin, and apigenin are the primary components of liquorice, demonstrating therapeutic effects against lung injury and pulmonary fibrosis. Liquiritin exhibits effective effectiveness in mitigating acute lung injury. Zhou et al. (2023) developed lung damage models utilising LPS in zebrafish and mice, revealing that Liquiritin markedly diminishes inflammation. This action may be ascribed to its suppression of the C-Jun N-terminal kinases (JNK)/Nur77/c-Jun signalling pathway. A separate study (Liu et al., 2020) indicated that the anti-acute lung injury activity of liquiritin may be associated with transient receptor potential vanilloid-1 (TRPV1), transient receptor potential A1 (TRPA1) targets, and the NF-κB signalling system. Research indicated that isoliquiritin apioside may safeguard the lungs by mitigating lung epithelial ferroptosis induced by hypoxia-inducible factor (Hif)-1α-mediated ferroptosis in the context of acute lung injury resulting from ischemia/reperfusion (II/R) (Zhongyin et al., 2022). Liquiritigenin is an active component of Gancao. Research indicates that liquiritigenin safeguards lung cells by dose-dependently suppressing the manufacture of α-hemolysin by *Staphylococcus aureus* (Dai et al., 2013). Liquiritigenin may provide an anti-pulmonary fibrosis effect, potentially through the modulation of the Sirtuin 1 (SIRT1)/Nrf2 signalling pathway to influence myofibroblast development (Hua and Ren, 2024). The preventive influence of glycyrrhizic acid on pulmonary function merits our consideration. Research indicates that glycyrrhizic acid can mitigate oxidative stress, diminish inflammation, and avert pulmonary fibrosis to improve lung damage caused by sodium nitrite (Elsherbini et al., 2021). The enhancement of lung function by glycyrrhizic acid is associated with the suppression of autophagy (Qu et al., 2019), apoptosis (Zhao et al., 2016), and immunological modulation (Ma et al., 2013). Apigenin is a flavonoid constituent of Gancao. Research indicates that apigenin safeguards lung function by modulating inflammatory responses, oxidative stress levels (Liu et al., 2018), correcting immunological imbalances, and reducing cellular apoptosis (Yu et al., 2023). Moreover, apigenin can mitigate the senescence of lung fibroblasts via the SIRT1-NAD-CD38 axis (Li J. et al., 2021).

### 5.5 Q-marker prediction based on metabolites measurability

TCM formulations comprise many TCM botanical drugs in specific ratios, making it essential to identify the principal metabolites that mediate their pharmacological effects. Therefore, Q-markers must be measurable.

Liu et al. (2018) employed the HPLC method to quantitatively assess the constituents of SLBZS, revealing concentrations of 3.12–3.29 mg/g for ginsenoside Rg1, 1.78–1.99 mg/g for Rb1, 1.65–1.82 mg/g for ginsenoside Re, 1.07–1.22 mg/g for liquiritin, and 4.55–4.89 mg/g for glycyrrhizic acid, respectively. This technique is exceptionally sensitive and specific, suitable for the quality control of SLBZS. Furthermore, Wang et al. (2024c) developed a quick determination method for ginsenosides Rb1, Rg1, and Re utilising near-infrared hyperspectral imaging (NIR-HSI) technology, thereby enhancing and supplementing the quality control method of SLBZS. A study by Xu et al. (2021) assessed the pharmacokinetics of the active constituents of SLBZS in rat serum, revealing that the serum concentrations of shendiol, ginsenoside Rg1, atractylenolide I, atractylenolide III, pachymic acid, neferine, nuciferine, diosgenin, platycodin D, and isoglycyrrhizin exhibited significant correlations (0.44–397.50, 0.63–388.50, 0.44–400.50, 0.54–490, 0.31–279.00, 0.41–367.50, 0.38–355.50, 0.50–447.00, 0.42–382.50, 0.39–356.10 ng/mL), which may serve as a benchmark for the quality control of SLBZS. A separate study (Ren et al., 2020) employed high-performance liquid chromatography with electrospray detection to quantify the concentrations of four components: liquiritinapioside, liquiritin, isoliquiritin apioside, and glycyrrhizic acid, yielding percentages of 0.26%–2.27%, 0.33%–5.07%, 0.14%–0.81%, and 0.77%–9.76%, respectively. Furthermore, quantitative analysis methods utilising HPLC for liquiritigenin, chikusetsusaponin V, and apigenin have been developed (Pei et al., 2019; Long et al., 2022; Yang et al., 2023).

In conclusion, according to the five principles established by Q-marker, the principal metabolites of SLBZS in the management of respiratory diseases include ginsenoside Rg1, ginsenoside Re, ginsenoside Rb1, atractylenolide I, atractonolide III, pachymic acid, Chikusetsusaponin V, liquiritin, isoliquiritin apioside, liquiritigenin, glycyrrhizic acid, and apigenin. Nonetheless, numerous issues persist in the study process concerning quality markers. 1) Constraints of metabolite detection methodologies. While UPLC-Q-TOF-MS/MS can swiftly identify numerous metabolites, its ability to differentiate isomers (e.g., ginsenoside Rg1 and Re) requires the integration of retention time or derivatisation techniques, and metabolites present in low concentrations may be overlooked due to inadequate ionisation efficiency. Moreover, certain blood-borne components may fail to achieve therapeutic quantities owing to limited protein binding or swift metabolism, whilst some undiscovered components may exert indirect effects via metabolism by gut flora. 2) Strengthen the hierarchical evaluation of data about component efficacy. The lung-protective action of ginsenoside Rg1 has only been confirmed in a murine model (Ji et al., 2021), and there is an absence of clinical randomised controlled trials to substantiate it. 3) Challenges in the practical implementation of quantifiable standards: HPLC-ELSD is appropriate for saponins lacking UV absorption (e.g., Chikusetsusaponin V) but exhibits lower sensitivity compared to LC-MS/MS; NIR-HSI is rapid yet necessitates a substantial sample size for calibration, potentially rendering it unsuitable for small batch production. The Q-marker prediction of SLBZS is hindered by the absence of *in vivo* target validation for most components through knockout experiments, inadequate data on pharmacokinetic interactions in compounding, and a deficiency of evidence-based medical research linking existing quality control criteria (e.g., glycyrrhizic acid) to clinical efficacy. This can be enhanced in the future by integrating web-based pharmacological prediction-experimental validation closed-loop studies with real-world data.

## 6 Conclusion

This study consolidated clinical records and established that SLBZS is useful in treating COPD, asthma, allergic rhinitis, *mycoplasma* pneumonia, recurrent respiratory tract infections, and other respiratory disorders. Pharmacological investigations concurrently demonstrate that SLBZS has advantages including the reduction of inflammation in lung tissue and airways, modulation of immunological responses, enhancement of gastrointestinal flora, and restoration of mitochondrial energy metabolism. This study aimed to elucidate the metabolites of SLBZS in the treatment of respiratory disorders by employing the five principles of Q-marker measurement to identify Q-markers relevant to SLBZS through five distinct criteria. In conclusion, twelve constituents, including ginsenoside Rg1, ginsenoside Re, ginsenoside Rb1, atractylenolide I, atractonolide III, pachymic acid, Chikusetsusaponin V, liquiritin, isoliquiritin apioside, liquiritigenin, glycyrrhizic acid, and apigenin, may function as Q-markers for SLBZS in the treatment of respiratory disorders. The clinical implementation of this formula encounters several challenges, such as non-standard randomisation, a restricted sample size, dosage imprecision, and insufficient monitoring of adverse effects. The pharmacological mechanism exhibits challenges such as unclear chemical ingredients, insufficient animal and cellular models, and an absence of thorough exploration of the mechanism of action. Moreover, there are challenges such as the lack of standardised research methodology for predicting quality markers and insufficient exploration of the relationship between quality markers. All of these difficulties have hindered the execution and progression of this method. Future endeavours should concentrate on broadening therapeutic uses, clarifying pharmacological mechanisms, and finding quality indicators.
